# *N*,*N*′-Dibenzyl-*N*′′-(2-chloro­acet­yl)-*N*,*N*′-dimethyl­phospho­ric triamide

**DOI:** 10.1107/S1600536811033204

**Published:** 2011-08-27

**Authors:** Mehrdad Pourayoubi, Mojtaba Keikha, Marek Nečas

**Affiliations:** a Department of Chemistry, Ferdowsi University of Mashhad, Mashhad, 91779, Iran; b Department of Chemistry, Faculty of Science, Masaryk University, Kotlarska 2, Brno CZ-61137, Czech Republic

## Abstract

In the title mol­ecule, C_18_H_23_ClN_3_O_2_P, the P atom is bonded in a distorted tetra­hedral environment. The P=O and N—H groups are *syn* with respect to each other. The angles at the tertiary N atoms confirm their *sp* ^2^ character. In the crystal, pairs of inter­molecular P=O⋯H—N hydrogen bonds form centrosymmetric dimers.

## Related literature

For background to compounds having a C(=O)NHP(=O) skeleton, see: Toghraee *et al.* (2011 ▶); Pourayoubi *et al.* (2011 ▶).
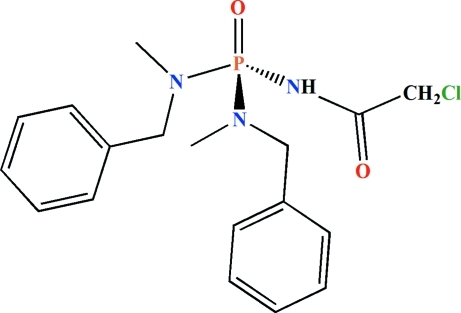


## Experimental

### 

#### Crystal data

C_18_H_23_ClN_3_O_2_P
*M* *_r_* = 379.81
Triclinic, 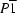

*a* = 9.5891 (8) Å
*b* = 9.9259 (7) Å
*c* = 10.2543 (8) Å
α = 89.509 (6)°
β = 74.945 (7)°
γ = 79.921 (6)°
*V* = 927.27 (12) Å^3^
*Z* = 2
Mo *K*α radiation
μ = 0.31 mm^−1^
*T* = 120 K
0.40 × 0.40 × 0.20 mm

#### Data collection

Oxford Diffraction Xcalibur Sapphire2 diffractometer
Absorption correction: multi-scan (*CrysAlis RED*; Oxford Diffraction, 2009 ▶) *T*_min_ = 0.983, *T*_max_ = 1.000
5838 measured reflections
3253 independent reflections
2594 reflections with *I* > 2σ(*I*)
*R*_int_ = 0.016

#### Refinement

*R*[*F* ^2^ > 2σ(*F* ^2^)] = 0.031
*wR*(*F* ^2^) = 0.083
*S* = 1.06
3253 reflections
232 parameters
H atoms treated by a mixture of independent and constrained refinement
Δρ_max_ = 0.25 e Å^−3^
Δρ_min_ = −0.37 e Å^−3^

### 

Data collection: *CrysAlis CCD* (Oxford Diffraction, 2009 ▶); cell refinement: *CrysAlis RED* (Oxford Diffraction, 2009 ▶); data reduction: *CrysAlis RED*; program(s) used to solve structure: *SHELXS97* (Sheldrick, 2008 ▶); program(s) used to refine structure: *SHELXL97* (Sheldrick, 2008 ▶); molecular graphics: *Mercury* (Macrae *et al.*, 2008 ▶); software used to prepare material for publication: *enCIFer* (Allen *et al.*, 2004 ▶).

## Supplementary Material

Crystal structure: contains datablock(s) I, global. DOI: 10.1107/S1600536811033204/lh5303sup1.cif

Structure factors: contains datablock(s) I. DOI: 10.1107/S1600536811033204/lh5303Isup2.hkl

Additional supplementary materials:  crystallographic information; 3D view; checkCIF report

## Figures and Tables

**Table 1:** Hydrogen-bond geometry (Å, °)

*D*—H⋯*A*	*D*—H	H⋯*A*	*D*⋯*A*	*D*—H⋯*A*
N1—H1*N*⋯O1^i^	0.773 (18)	2.037 (19)	2.795 (2)	167.1 (19)

Symmetry code: (i) 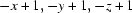.

**Fig. 1.**
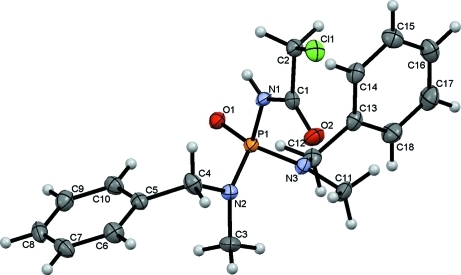
The molecular structure of the title compound with ellipsoids shown at the 50% probability level.

## supplementary crystallographic information

### Comment

The structure determination of the title compound was performed as part of a project in our laboratory on the synthesis of new phosphoramidates with formula RC(O)NHP(O)[N*R*'*R*"]_2_ (Toghraee *et al.*, 2011). The molecular structure of the title compound is shown in Fig. 1. The P1—N2 and P1—N3 bonds are shorter than the P1—N1 bond. The *sp*^2^ character of the tertiary N atoms is indicated by the angles around N2 and N3. The C1—N1—P1 angle is the most distorted from the expected 120° (in terms of *sp*^2^ hybridization). The P═O bond length is standard for this type of phosphoramidate compounds (Pourayoubi *et al.*, 2011). The hydrogen atom of the C(═O)NHP(═O) group is involved in an intermolecular –P═O···H—N– hydrogen bond (Table 1). Pair of this type of hydrogen bond forms centrosymmetric dimer which is the usual H-bond pattern for compounds with the general formula RC(O)NHP(O)[N*R*'*R*"]_2_, where *R*' and *R*" ≠ H, in the case of *syn* orientation of P═O *versus* N—H (Toghraee *et al.*, 2011).

### Experimental

Synthesis of CH_2_ClC(O)NHP(O)Cl_2_: The reaction of phosphorus pentachloride (20 mmol) and 2-chloroacetamide (20 mmol) in dry benzene (40 ml) at 353 K (3 h) and then the treatment of formic acid 85% (20 mmol) at room temperature leads to the formation of CH_2_ClC(O)NHP(O)Cl_2_ as solid product.

Synthesis of title compound: To a solution of (3.47 mmol) CH_2_ClC(O)NHP(O)Cl_2_ in CHCl_3_ (25 ml), a solution of *N*-methylbenzylamine (13.88 mmol) in CHCl_3_ (5 ml) was added dropwise at 273 K. After 6 h of stirring, the solvent was evaporated in vacuum. The solid was washed with distilled water. Single crystals were obtained from a solution of the title compound in chloroform and n-heptane after slow evaporation at room temperature. IR (KBr, cm^-1^): 3116, 2920, 1728, 1494, 1341, 1193, 1011, 949, 867, 800, 743, 695.

### Refinement

All carbon bound H atoms were placed in calculated positions and were refined as riding with their *U*_iso_ set to either 1.2*U*_eq_ or 1.5*U*_eq_ (methyl) of the respective carrier atoms. The N bound H atom was located in a difference Fourier map and refined isotropically.

### Crystal data

**Table:**

C_18_H_23_ClN_3_O_2_P	*Z* = 2
*M**_r_* = 379.81	*F*(000) = 400
Triclinic, *P*1	*D*_x_ = 1.360 Mg m^−^^3^
Hall symbol: -P 1	Mo *K*α radiation, λ = 0.71073 Å
*a* = 9.5891 (8) Å	Cell parameters from 3547 reflections
*b* = 9.9259 (7) Å	θ = 3.1–27.6°
*c* = 10.2543 (8) Å	µ = 0.31 mm^−^^1^
α = 89.509 (6)°	*T* = 120 K
β = 74.945 (7)°	Plate, colorless
γ = 79.921 (6)°	0.40 × 0.40 × 0.20 mm
*V* = 927.27 (12) Å^3^	

### Data collection

**Table:**

Oxford Diffraction Xcalibur Sapphire2 diffractometer	3253 independent reflections
Radiation source: Enhance (Mo) X-ray Source	2594 reflections with *I* > 2σ(*I*)
graphite	*R*_int_ = 0.016
Detector resolution: 8.4353 pixels mm^-1^	θ_max_ = 25.0°, θ_min_ = 3.1°
ω scans	*h* = −11→11
Absorption correction: multi-scan (*CrysAlis RED*; Oxford Diffraction, 2009)	*k* = −11→10
*T*_min_ = 0.983, *T*_max_ = 1.000	*l* = −12→11
5838 measured reflections	

### Refinement

**Table:**

Refinement on *F*^2^	Primary atom site location: structure-invariant direct methods
Least-squares matrix: full	Secondary atom site location: difference Fourier map
*R*[*F*^2^ > 2σ(*F*^2^)] = 0.031	Hydrogen site location: inferred from neighbouring sites
*wR*(*F*^2^) = 0.083	H atoms treated by a mixture of independent and constrained refinement
*S* = 1.06	*w* = 1/[σ^2^(*F*_o_^2^) + (0.0449*P*)^2^ + 0.0312*P*] where *P* = (*F*_o_^2^ + 2*F*_c_^2^)/3
3253 reflections	(Δ/σ)_max_ < 0.001
232 parameters	Δρ_max_ = 0.25 e Å^−^^3^
0 restraints	Δρ_min_ = −0.37 e Å^−^^3^

### Special details

**Table:**

Geometry. All e.s.d.'s (except the e.s.d. in the dihedral angle between two l.s. planes) are estimated using the full covariance matrix. The cell e.s.d.'s are taken into account individually in the estimation of e.s.d.'s in distances, angles and torsion angles; correlations between e.s.d.'s in cell parameters are only used when they are defined by crystal symmetry. An approximate (isotropic) treatment of cell e.s.d.'s is used for estimating e.s.d.'s involving l.s. planes.
Refinement. Refinement of *F*^2^ against ALL reflections. The weighted *R*-factor *wR* and goodness of fit *S* are based on *F*^2^, conventional *R*-factors *R* are based on *F*, with *F* set to zero for negative *F*^2^. The threshold expression of *F*^2^ > σ(*F*^2^) is used only for calculating *R*-factors(gt) *etc*. and is not relevant to the choice of reflections for refinement. *R*-factors based on *F*^2^ are statistically about twice as large as those based on *F*, and *R*- factors based on ALL data will be even larger.

### Fractional atomic coordinates and isotropic or equivalent isotropic displacement parameters (Å^2^)

**Table:**

	*x*	*y*	*z*	*U*_iso_*/*U*_eq_	
Cl1	0.35277 (5)	0.03423 (5)	0.36846 (5)	0.02726 (14)	
P1	0.73295 (5)	0.38427 (4)	0.38957 (5)	0.01865 (13)	
O1	0.68787 (12)	0.51744 (12)	0.46676 (12)	0.0219 (3)	
O2	0.64902 (13)	0.10825 (13)	0.30353 (13)	0.0287 (3)	
N1	0.58216 (17)	0.31087 (16)	0.42742 (16)	0.0201 (4)	
N2	0.78048 (15)	0.38421 (15)	0.22483 (14)	0.0219 (3)	
N3	0.87044 (15)	0.29050 (14)	0.43213 (15)	0.0216 (3)	
C1	0.55954 (19)	0.18865 (18)	0.38376 (18)	0.0205 (4)	
C2	0.40604 (18)	0.16286 (18)	0.45401 (18)	0.0217 (4)	
H2A	0.3347	0.2491	0.4595	0.026*	
H2B	0.4035	0.1350	0.5474	0.026*	
C3	0.9309 (2)	0.3970 (2)	0.15080 (19)	0.0318 (5)	
H3A	0.9552	0.3517	0.0611	0.048*	
H3B	0.9999	0.3536	0.2009	0.048*	
H3C	0.9378	0.4941	0.1410	0.048*	
C4	0.6704 (2)	0.42144 (19)	0.14777 (19)	0.0275 (5)	
H4A	0.5718	0.4206	0.2087	0.033*	
H4B	0.6879	0.3508	0.0750	0.033*	
C5	0.67105 (19)	0.55997 (18)	0.08528 (18)	0.0220 (4)	
C6	0.6768 (2)	0.5718 (2)	−0.05137 (19)	0.0279 (4)	
H6A	0.6827	0.4925	−0.1052	0.033*	
C7	0.6740 (2)	0.6988 (2)	−0.1093 (2)	0.0298 (5)	
H7A	0.6778	0.7057	−0.2026	0.036*	
C8	0.6658 (2)	0.8148 (2)	−0.0331 (2)	0.0292 (5)	
H8A	0.6639	0.9014	−0.0735	0.035*	
C9	0.6604 (2)	0.80430 (19)	0.1031 (2)	0.0292 (5)	
H9A	0.6551	0.8838	0.1564	0.035*	
C10	0.6627 (2)	0.67757 (19)	0.16120 (19)	0.0256 (4)	
H10A	0.6585	0.6711	0.2546	0.031*	
C11	0.9385 (2)	0.15360 (18)	0.37333 (19)	0.0292 (5)	
H11A	1.0446	0.1490	0.3388	0.044*	
H11B	0.8972	0.1342	0.2990	0.044*	
H11C	0.9193	0.0858	0.4427	0.044*	
C12	0.9306 (2)	0.33736 (19)	0.53760 (19)	0.0259 (4)	
H12A	0.8888	0.4355	0.5596	0.031*	
H12B	1.0381	0.3295	0.5013	0.031*	
C13	0.90090 (19)	0.25971 (17)	0.66624 (18)	0.0224 (4)	
C14	0.7603 (2)	0.23941 (18)	0.73019 (19)	0.0250 (4)	
H14A	0.6822	0.2705	0.6901	0.030*	
C15	0.7324 (2)	0.1745 (2)	0.85159 (19)	0.0296 (5)	
H15A	0.6353	0.1621	0.8946	0.035*	
C16	0.8448 (2)	0.12755 (19)	0.9108 (2)	0.0318 (5)	
H16A	0.8254	0.0829	0.9943	0.038*	
C17	0.9843 (2)	0.1458 (2)	0.8484 (2)	0.0369 (5)	
H17A	1.0621	0.1133	0.8884	0.044*	
C18	1.0130 (2)	0.2115 (2)	0.7269 (2)	0.0316 (5)	
H18A	1.1103	0.2237	0.6846	0.038*	
H1N	0.512 (2)	0.359 (2)	0.4672 (18)	0.022 (6)*	

### Atomic displacement parameters (Å^2^)

**Table:**

	*U*^11^	*U*^22^	*U*^33^	*U*^12^	*U*^13^	*U*^23^
Cl1	0.0285 (3)	0.0242 (3)	0.0337 (3)	−0.0101 (2)	−0.0129 (2)	0.0023 (2)
P1	0.0146 (2)	0.0162 (2)	0.0238 (3)	−0.00197 (18)	−0.00340 (19)	0.00312 (19)
O1	0.0177 (6)	0.0183 (7)	0.0288 (7)	−0.0027 (5)	−0.0051 (5)	0.0008 (5)
O2	0.0228 (7)	0.0233 (7)	0.0354 (8)	−0.0023 (6)	−0.0007 (6)	−0.0064 (6)
N1	0.0138 (8)	0.0174 (8)	0.0251 (9)	−0.0002 (7)	−0.0001 (7)	−0.0008 (7)
N2	0.0179 (8)	0.0227 (8)	0.0231 (9)	−0.0024 (6)	−0.0027 (7)	0.0048 (7)
N3	0.0183 (8)	0.0197 (8)	0.0257 (9)	0.0001 (6)	−0.0065 (7)	0.0025 (7)
C1	0.0223 (10)	0.0184 (9)	0.0220 (10)	−0.0026 (8)	−0.0089 (8)	0.0037 (8)
C2	0.0212 (9)	0.0183 (9)	0.0262 (10)	−0.0055 (8)	−0.0058 (8)	−0.0006 (8)
C3	0.0243 (10)	0.0355 (12)	0.0280 (11)	−0.0028 (9)	0.0045 (9)	0.0054 (9)
C4	0.0355 (11)	0.0262 (11)	0.0250 (11)	−0.0104 (9)	−0.0123 (9)	0.0041 (8)
C5	0.0189 (9)	0.0246 (10)	0.0247 (10)	−0.0065 (8)	−0.0079 (8)	0.0055 (8)
C6	0.0298 (11)	0.0282 (11)	0.0286 (11)	−0.0089 (9)	−0.0105 (9)	0.0022 (9)
C7	0.0293 (11)	0.0388 (12)	0.0250 (11)	−0.0118 (9)	−0.0102 (9)	0.0123 (9)
C8	0.0244 (10)	0.0255 (11)	0.0408 (12)	−0.0080 (8)	−0.0122 (9)	0.0145 (9)
C9	0.0256 (10)	0.0230 (10)	0.0394 (12)	−0.0051 (8)	−0.0089 (9)	0.0007 (9)
C10	0.0265 (10)	0.0292 (11)	0.0228 (10)	−0.0071 (8)	−0.0083 (8)	0.0046 (8)
C11	0.0225 (10)	0.0228 (10)	0.0387 (12)	0.0046 (8)	−0.0074 (9)	0.0007 (9)
C12	0.0184 (9)	0.0256 (10)	0.0366 (12)	−0.0074 (8)	−0.0099 (9)	0.0056 (9)
C13	0.0228 (9)	0.0163 (9)	0.0299 (11)	−0.0035 (8)	−0.0100 (8)	−0.0027 (8)
C14	0.0232 (10)	0.0232 (10)	0.0320 (11)	−0.0049 (8)	−0.0125 (9)	0.0010 (9)
C15	0.0289 (11)	0.0309 (11)	0.0315 (12)	−0.0121 (9)	−0.0079 (9)	0.0009 (9)
C16	0.0416 (12)	0.0290 (11)	0.0285 (11)	−0.0089 (9)	−0.0140 (10)	0.0039 (9)
C17	0.0339 (12)	0.0396 (13)	0.0404 (13)	0.0013 (10)	−0.0205 (10)	0.0046 (10)
C18	0.0217 (10)	0.0382 (12)	0.0363 (12)	−0.0041 (9)	−0.0109 (9)	0.0037 (10)

### Geometric parameters (Å, °)

**Table:**

Cl1—C2	1.7723 (17)	C7—C8	1.376 (3)
P1—O1	1.4836 (12)	C7—H7A	0.9500
P1—N3	1.6302 (14)	C8—C9	1.388 (3)
P1—N2	1.6313 (15)	C8—H8A	0.9500
P1—N1	1.6866 (15)	C9—C10	1.387 (3)
O2—C1	1.206 (2)	C9—H9A	0.9500
N1—C1	1.368 (2)	C10—H10A	0.9500
N1—H1N	0.773 (18)	C11—H11A	0.9800
N2—C3	1.472 (2)	C11—H11B	0.9800
N2—C4	1.472 (2)	C11—H11C	0.9800
N3—C11	1.461 (2)	C12—C13	1.510 (2)
N3—C12	1.465 (2)	C12—H12A	0.9900
C1—C2	1.526 (2)	C12—H12B	0.9900
C2—H2A	0.9900	C13—C14	1.386 (2)
C2—H2B	0.9900	C13—C18	1.390 (3)
C3—H3A	0.9800	C14—C15	1.382 (2)
C3—H3B	0.9800	C14—H14A	0.9500
C3—H3C	0.9800	C15—C16	1.381 (3)
C4—C5	1.512 (2)	C15—H15A	0.9500
C4—H4A	0.9900	C16—C17	1.367 (3)
C4—H4B	0.9900	C16—H16A	0.9500
C5—C10	1.388 (2)	C17—C18	1.387 (3)
C5—C6	1.393 (2)	C17—H17A	0.9500
C6—C7	1.388 (3)	C18—H18A	0.9500
C6—H6A	0.9500		
			
O1—P1—N3	110.79 (7)	C8—C7—C6	120.72 (18)
O1—P1—N2	118.78 (7)	C8—C7—H7A	119.6
N3—P1—N2	105.79 (8)	C6—C7—H7A	119.6
O1—P1—N1	105.09 (7)	C7—C8—C9	119.47 (17)
N3—P1—N1	111.96 (8)	C7—C8—H8A	120.3
N2—P1—N1	104.33 (8)	C9—C8—H8A	120.3
C1—N1—P1	130.67 (14)	C10—C9—C8	119.86 (18)
C1—N1—H1N	114.7 (14)	C10—C9—H9A	120.1
P1—N1—H1N	113.9 (14)	C8—C9—H9A	120.1
C3—N2—C4	114.46 (15)	C9—C10—C5	121.12 (18)
C3—N2—P1	120.68 (13)	C9—C10—H10A	119.4
C4—N2—P1	121.30 (12)	C5—C10—H10A	119.4
C11—N3—C12	115.28 (14)	N3—C11—H11A	109.5
C11—N3—P1	123.34 (12)	N3—C11—H11B	109.5
C12—N3—P1	121.34 (12)	H11A—C11—H11B	109.5
O2—C1—N1	125.44 (16)	N3—C11—H11C	109.5
O2—C1—C2	123.36 (15)	H11A—C11—H11C	109.5
N1—C1—C2	111.16 (15)	H11B—C11—H11C	109.5
C1—C2—Cl1	112.44 (12)	N3—C12—C13	114.54 (14)
C1—C2—H2A	109.1	N3—C12—H12A	108.6
Cl1—C2—H2A	109.1	C13—C12—H12A	108.6
C1—C2—H2B	109.1	N3—C12—H12B	108.6
Cl1—C2—H2B	109.1	C13—C12—H12B	108.6
H2A—C2—H2B	107.8	H12A—C12—H12B	107.6
N2—C3—H3A	109.5	C14—C13—C18	118.12 (18)
N2—C3—H3B	109.5	C14—C13—C12	121.04 (16)
H3A—C3—H3B	109.5	C18—C13—C12	120.79 (16)
N2—C3—H3C	109.5	C15—C14—C13	120.82 (17)
H3A—C3—H3C	109.5	C15—C14—H14A	119.6
H3B—C3—H3C	109.5	C13—C14—H14A	119.6
N2—C4—C5	114.34 (14)	C16—C15—C14	120.40 (18)
N2—C4—H4A	108.7	C16—C15—H15A	119.8
C5—C4—H4A	108.7	C14—C15—H15A	119.8
N2—C4—H4B	108.7	C17—C16—C15	119.44 (19)
C5—C4—H4B	108.7	C17—C16—H16A	120.3
H4A—C4—H4B	107.6	C15—C16—H16A	120.3
C10—C5—C6	118.48 (16)	C16—C17—C18	120.46 (18)
C10—C5—C4	121.77 (16)	C16—C17—H17A	119.8
C6—C5—C4	119.73 (17)	C18—C17—H17A	119.8
C7—C6—C5	120.34 (18)	C17—C18—C13	120.75 (18)
C7—C6—H6A	119.8	C17—C18—H18A	119.6
C5—C6—H6A	119.8	C13—C18—H18A	119.6
			
O1—P1—N1—C1	178.95 (16)	N2—C4—C5—C6	129.08 (17)
N3—P1—N1—C1	−60.70 (18)	C10—C5—C6—C7	−0.1 (3)
N2—P1—N1—C1	53.24 (18)	C4—C5—C6—C7	178.50 (17)
O1—P1—N2—C3	83.81 (15)	C5—C6—C7—C8	0.1 (3)
N3—P1—N2—C3	−41.38 (15)	C6—C7—C8—C9	0.0 (3)
N1—P1—N2—C3	−159.63 (13)	C7—C8—C9—C10	−0.3 (3)
O1—P1—N2—C4	−73.72 (15)	C8—C9—C10—C5	0.3 (3)
N3—P1—N2—C4	161.09 (13)	C6—C5—C10—C9	−0.1 (3)
N1—P1—N2—C4	42.84 (15)	C4—C5—C10—C9	−178.68 (17)
O1—P1—N3—C11	179.82 (13)	C11—N3—C12—C13	−67.5 (2)
N2—P1—N3—C11	−50.20 (15)	P1—N3—C12—C13	110.20 (16)
N1—P1—N3—C11	62.85 (15)	N3—C12—C13—C14	−50.5 (2)
O1—P1—N3—C12	2.28 (15)	N3—C12—C13—C18	132.22 (18)
N2—P1—N3—C12	132.27 (13)	C18—C13—C14—C15	0.8 (3)
N1—P1—N3—C12	−114.68 (13)	C12—C13—C14—C15	−176.61 (16)
P1—N1—C1—O2	−1.4 (3)	C13—C14—C15—C16	−0.6 (3)
P1—N1—C1—C2	176.42 (13)	C14—C15—C16—C17	0.0 (3)
O2—C1—C2—Cl1	−19.1 (2)	C15—C16—C17—C18	0.3 (3)
N1—C1—C2—Cl1	162.99 (12)	C16—C17—C18—C13	−0.1 (3)
C3—N2—C4—C5	−51.7 (2)	C14—C13—C18—C17	−0.4 (3)
P1—N2—C4—C5	107.18 (16)	C12—C13—C18—C17	176.96 (18)
N2—C4—C5—C10	−52.4 (2)		

### Hydrogen-bond geometry (Å, °)

**Table:**

*D*—H···*A*	*D*—H	H···*A*	*D*···*A*	*D*—H···*A*
N1—H1N···O1^i^	0.773 (18)	2.037 (19)	2.795 (2)	167.1 (19)

Symmetry codes: (i) −*x*+1, −*y*+1, −*z*+1.
